# Prerequisites for an Artificial Self

**DOI:** 10.3389/fnbot.2020.00005

**Published:** 2020-02-21

**Authors:** Verena V. Hafner, Pontus Loviken, Antonio Pico Villalpando, Guido Schillaci

**Affiliations:** ^1^Adaptive Systems Group, Computer Science Department, Humboldt-Universität zu Berlin, Berlin, Germany; ^2^Softbank Robotics, Paris, France; ^3^Centre for Robotics and Neural Systems (CRNS), University of Plymouth, Plymouth, United Kingdom; ^4^The BioRobotics Institute, Scuola Superiore Sant'Anna, Pisa, Italy; ^5^Department of Excellence in Robotics & AI, Scuola Superiore Sant'Anna, Pisa, Italy

**Keywords:** artificial self, developmental robotics, sense of agency, predictive processes, sense of body ownership, minimal self

## Abstract

Traditionally investigated in philosophy, body ownership and agency—two main components of the minimal self—have recently gained attention from other disciplines, such as brain, cognitive and behavioral sciences, and even robotics and artificial intelligence. In robotics, intuitive human interaction in natural and dynamic environments becomes more and more important, and requires skills such as self-other distinction and an understanding of agency effects. In a previous review article, we investigated studies on mechanisms for the development of motor and cognitive skills in robots (Schillaci et al., 2016). In this review article, we argue that these mechanisms also build the foundation for an understanding of an artificial self. In particular, we look at developmental processes of the minimal self in biological systems, transfer principles of those to the development of an artificial self, and suggest metrics for agency and body ownership in an artificial self.

## 1. Introduction

People can usually easily recognize their own body and the results of their own actions. This apparently simple skill likely contributes to what makes us feel as separate entities in the world (Van Den Bos and Jeannerod, 2002) and it is indeed fundamental for interacting with the environment and with other individuals. A current research trend suggests that the *minimal self*- the pre-reflective experience of being a self, or the awareness of oneself as a subject of experience (Blanke and Metzinger, 2009)— would be characterized by two important aspects: a sense of body ownership—*I feel corporal sensations as uniquely belonging to my own body*—and a sense of agency - *I feel being in control of my own actions* (Gallagher, 2000).

Topics such as body ownership and agency that have traditionally been investigated in philosophy have recently gained attention from other disciplines, such as brain, cognitive and behavioral sciences, and even robotics and artificial intelligence. Some neuroscientists, for example, interpret certain human mental disorders—such as schizophrenia—as the result of a disrupted sense of the self (Frith et al., 2000; Nelson et al., 2014; Klaver and Dijkerman, 2016; Sterzer et al., 2016). In robotics, intuitive human interaction in natural and dynamic environments becomes more and more important, and requires skills such as self-other distinction and an understanding of agency effects (Holthaus and Wachsmuth, 2012; Belpaeme et al., 2018). Developmental psychologists study the emergence of self-awareness from very early stages of development. Self-awareness would unfold already during the first months of life, when infants seem to start having a sense of how their own body is situated in relation to other entities in the environment (Rochat, 2003). Infants at 5 months of age, for example, are able to distinguish their own leg movements from those of another infant, when they are displayed in a mirror (Rochat, 2003). These action-effects have been studied in infants using different modalities including sound (Paulus et al., 2012).

These findings represent a valuable source of inspiration for roboticists, whose aim is to develop autonomous robots capable of living in and interacting with the human society. Developmental robotics addresses this challenge by implementing methods and algorithms for motor and cognitive development in artificial systems inspired by infant development (Cangelosi and Schlesinger, 2015). In developmental robotics, state of the art machine learning techniques are applied to computational models, creating artificial systems that can adapt to new situations and learn in an open-ended fashion. The emergence of the self represents a key step in cognitive development. Therefore, there is a growing interest in the developmental robotics community on implementing processes capable of enabling the experience of the self—with phenomena such as sense of body ownership and agency—in artificial agents.

On the other side, robots can represent valuable tools to investigate phenomena of subjective experience typical of humans. In fact, robots are equipped with sensors and actuators that can be inspected and controlled during their operations. What the robot sees and perceives, and its internal states can be logged and further analyzed which is obviously not possible in humans. If robots were capable of detecting and recognizing their own body and movements, their interaction with the environment and with people would be much more efficient and natural. However, the questions about which computational processes are needed to implement a primitive sense of body ownership and agency in robots, and of how the ontogenetic process of the individual shapes the development of the self, are still open.

This manuscript follows-up a previous review paper (Schillaci et al., 2016), in which we investigated studies on mechanisms for the development of motor and cognitive skills in robots. In this review paper, we argue that the same mechanisms also build the foundation for the development of an artificial self. In fact, in infants, the self seems to emerge along the motor and cognitive development of the individual (Lagercrantz and Changeux, 2009). Implementing similar processes in artificial systems may provide insights also in the possibility to develop an artificial self. In this work, we address the role of developmental processes in the emergence of an artificial self, and we suggest the concept of *self-manifolds* in artificial systems and the use of metrics for establishing the boundaries of an artificial self.

The review paper is structured as follows. First, in section 2, we revisit the concepts addressed in our previous review (Schillaci et al., 2016) and frame them within the context of the development of an artificial self. In particular, we present advances in the study of behavioral and computational components that allow autonomous motor and cognitive development in artificial systems. We discuss how these components can build the foundation for an artificial self. In order to do so, we ask whether and how the minimal self is affected during the ontogenetic process of the individual, and how open-ended learning and social interaction can shape the development of an artificial self, and then review robotic studies addressing this question. In section 3, we review studies on metrics and boundaries of the human self, and propose their use also for artificial systems. Finally, in section 4, we provide our conclusions and open challenges in the quest for the development of an artificial self.

## 2. Behavioral and Computational Components

In the robotics literature, the study on the artificial minimal self is young and fragmented. Unfortunately, a study presenting a comprehensive overview on the robotic investigations on this topic is missing. Nonetheless, many articles can be found providing interesting insights on aspects and prerequisites that can be related to the development of an artificial self. Two recent papers highlight both aspects of the human minimal self and an artificial minimal self. Georgie et al. (2019) look at developmental indices and behavioral measures of the minimal self, and Lanillos et al. (2019) look into computational models of neurological disorders related to the minimal self. In particular, they look into the balance between sensed and predicted sensory effects in ASD and schizophrenia.

In a previous review paper (Schillaci et al., 2016), we investigated studies on mechanisms for the development of motor and cognitive skills in robots. In particular, we identified three main behavioral and computational components that can enable autonomous acquisition of motor skills and the implementation of basic cognitive capabilities: (1) exploration behaviors; (2) internal body representations; (3) sensorimotor simulations. In this review, we extend the review provided in Schillaci et al. (2016) by creating links to the topic of the development of an artificial self, beside introducing more recent robotic studies on related topics. We particularly focus on those ones that propose strategies to scale up with motor and cognitive development. We extend exploration behaviors with artificial curiosity and sensorimotor simulations with predictive processes in order to strengthen the aspects of the development of a minimal self. All three components are processes or cognitive skills that run in parallel and independently from each other and can be seen as building blocks of the minimal self as discussed later.

### 2.1. Self-Exploration Behaviors and Artificial Curiosity

Human fetuses seem to already have some limited control on their body, as they react to touch, sound, smell, and pain, and even show facial expressions responding to external stimuli (Lowery et al., 2007). Some researchers (Lagercrantz and Changeux, 2009), though, believe that these reactions may have subcortical non-conscious origin and that, only shortly after birth, newborns show signs of basic self-awareness. In fact, developmental studies provide evidence about infant behaviors displaying some level of self-awareness in their first weeks of life (Rochat, 2011). Nonetheless, whether—and to what extend—self-awareness is present at birth, developmental researchers believe that it would unfold during early stages of development [see Rochat (2003) for empirical evidence and proposals]. However, why and how self-awareness exactly would emerge during infancy are still open questions and in particular there are no thorough theories or computational models explaining their function. Hart and Scassellati (2011) argue that self-identification algorithms are the first step toward a more comprehensive model of the robotic self.

There is a general consensus on recognizing the important role in the development of self-awareness to the perceptual experiences that toddlers undergo when exploring and playing with their surroundings. The self would emerge through the active interaction with one's physical and social environment (Verschoor and Hommel, 2017). Indeed, exploration behaviors are recognized as the means for motor and cognitive development in infants, as well as in robots [see Schillaci et al. (2016) for a review]. Several studies investigate the cognitive mechanisms and drives behind exploration and play in infancy. In infants, curiosity—which is usually inferred through their use of prolonged visual attention to stimuli (Benson and Haith, 2010. p. 157–167; Grgič et al., 2016) is thought to drive the emergence of ordered developmental trajectories, including in domains such as vocal development, imitation and tool use discovery (Acevedo-Valle et al., 2018; Oudeyer, 2018). This is contrary to earlier belief that infants learn by random actions, but rather that their actions are goal-directed from the very start (Von Hofsten, 2004).

Infants' curiosity, play and exploration—and the likely goal-directed nature of their actions—have attracted the interest of developmental roboticists. In fact, studies on artificial curiosity have demonstrated how mechanisms for goal-directed exploration can be used to efficiently learn robot dynamics, even if the artificial system is characterized by complex high-dimensional embodiments. Artificial curiosity goes beyond novelty detection that would drive the agent to novel, but not necessarily predictable regions of its sensorimotor space. In contrast, artificial curiosity drives the agent toward regions where the learning progress can be maximized (Oudeyer et al., 2007). The main difference to typical machine learning scenarios is that the agent creates its own training samples for a desired learning trajectory.

The first studies on artificial curiosity and exploration in robots were limited, in a way. Although promising and demonstrating that curiosity-driven and exploration behaviors can efficiently solve inverse and forward kinematics problems, they mostly focused on relatively simple tasks, such as reaching actions for robot manipulators. Prolonged and incremental learning, until recently, was not a main priority in these studies. Indeed, it is still a great challenge in the whole robotics community. Seemingly, assuming that, in infants, self-awareness is a result of complex and prolonged interactions and experiences, the study on the development of an artificial self has to address, as well, how self-awareness would unfold along incremental learning in robots.

**Figure 1:**
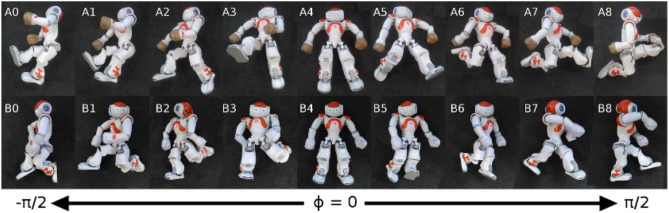
Curiosity-based learning method for humanoid robots using postures and regions. This image shows an example of postures learned after 30 min of online learning (Loviken et al., 2018). **(A,B)** Represent two independent runs, and the number indicate the state. Each state is responsible for an interval of angle ϕ, where ϕ is the torso's orientation in relation to the ground. A demonstration video can be found at this URL: https://www.youtube.com/watch?v=QzZsJxyGGIk.

Recently, interesting studies have been published on topics close to this line of thoughts. For instance, studies in the literature on goal-directed exploration in artificial systems proposed ways to scale up learning to multiple task spaces (Forestier and Oudeyer, 2016; Forestier et al., 2017) or to domains where exploration of a task space requires action planning in multiple steps (Loviken and Hemion, 2017; Loviken et al., 2018). Figure 1 shows the results of a curiosity-based learning method for humanoid robots, where the sensory space was partitioned into a disjoint set of finite elements. In this space, every element was seen as an independent goal-babbling problem and a planning module could be added by observing transitions between the different elements (Loviken and Hemion, 2017; Loviken et al., 2018).

Acevedo-Valle et al. (2018) studied intrinsic motivation systems in the context of early vocal development which further develop through social reinforcement. An artificial agent was endowed with a proprioceptive mechanism, which was used to prevent the execution of unreachable motor configurations or invalid (painful) configurations. Moreover, the authors introduced an expert instructor which produced correct utterances whenever the exploring autonomous learner was emitting similar (although still not correct) sounds. This resulted in a social reinforcement, which provided clues to the learner of interesting sensorimotor regions to explore.

Interesting advances have been made also in the context of goal generation. For instance, Mannella et al. (2018) show how an artificial system can autonomously generate goals to be used in an intrinsic motivation system to explore and to gather knowledge about its own body. In Schillaci et al. (2020), the authors present an architecture for curiosity-driven goal-directed exploration behaviors on a camera-equipped robot arm. A combination of deep neural networks for offline unsupervised learning of low-dimensional features from images, and of online learning of shallow neural networks was used. The artificial curiosity system assigned interest values to a set of pre-defined goals, and drove the exploration toward those that were expected to maximize the learning progress. Moreover, the authors proposed the integration of an episodic memory system to face catastrophic forgetting issues, typically experienced when performing online updates of artificial neural networks. The results showed that adopting an episodic memory system not only prevented the computational models from quickly forgetting knowledge that have been previously acquired, but also provided new avenues for modulating the balance between plasticity and stability of the models.

In humans, the self develops along the ontogenetic process of the individual. This is closely related to mechanisms of open-ended learning and social interaction, but also on the establishment and refinement of plastic body representations. The next section will provide an overview of recent studies on body representations in artificial systems.

### 2.2. Body Representations

Many researchers have suggested theories in trying to explain the experience of body ownership and agency, and self-awareness in general. Sense of agency and sense of body ownership seem to be strongly linked, but many empirical studies still investigate them separately from each other. The appearance of the first signs of self-awareness in newborns seems to be dependent to the establishment of thalamocortical connections (Lagercrantz and Changeux, 2009). In general, the sense of body ownership seems to be strongly intertwined with an internal representation of the body maintained by our brain. Here we adopt the conceptual clarification by Gallagher (1986) between *body image* and *body schema*, where body image is a conscious representation or image of the body, whereas body schema is a non-conscious representation of sensorimotor skills. While we interact with the environment, we generate a rich set of multi-modal sensory and motor experience (Schillaci et al., 2016). This information has been proposed to be integrated in a sort of a body schema into our brain, which would keep an up-to-date representation of the positions and configurations of the different body parts in space (Maravita et al., 2003; Hoffmann et al., 2010). Moreover, the body schema very likely undergoes a continuous process of adaptation, as humans and animals follow an ontogenetic process where corporal dimensions and morphology change over time. The way in which we represent and feel our body seems to strongly rely on these representations, which would integrate inputs from different sensory modalities (Azañón et al., 2016). Scientists carried out experiments to explore how the brain combines information from the flow of sensory input data to create a feeling of body ownership, such as the famous experiment of the rubber hand illusion, where the participant is confused by the sight of a fake hand and synchronized sensory stimulation (Botvinick and Cohen, 1998).

Some researchers in cognitive development link the construction of the self to the experience encoded in a sort of autobiographical memory (Nelson, 2003). Pointeau and Dominey (2017) review a range of robotic experiments that address different aspects of the self and relate them to the definition of the self as given by Neisser (1995). Ulric Neisser proposed five types of self-knowledge that correspond to five distinct components of the self: ecological, interpersonal, conceptual, temporally extended, and private. The *ecological self* , that is “the individual situated in and acting upon the immediate physical environment” (Neisser, 1995), is perhaps the level which is most interesting here, and it is rather easy, given the current robot technologies, to design robotic experiments addressing it. Ecological proprioception is integrated with different modalities of sensory information concerning one's own body as interacting within the environment (Gallagher, 2007). The tactile modality has received particular interest from researchers on subjective experiences, and on their impairments in patients with brain disorders. Van Stralen et al. (2011), for instance, studied how self-touch influences the structural representation of one's own body and found that self-touch may be modulating impairments in body ownership.

Developmental roboticists have also focused their attention onto the role of the tactile modality in the formation and maintenance of body representations. For instance, Zenha et al. (2018) studied how a body schema can be adapted incrementally in a humanoid robot based on touch events. Hoffmann (2017) studied the role of self-touch experiences in the formation of a self. Self-touch would provide redundant information that would facilitate the formation of a body representation. Timing and synchrony has been identified also as an important feature in support to the integration of information from multiple modalities within a body representations. Nabeshima et al. (2005) present a robotic study in support of that.

Hoffmann et al. (2018) studied a self-organizing model for body representation on an iCub humanoid robot with an artificial pressure-sensitive skin. In particular, the proposed framework was used to learn a topographic representation of the robot's body surface from experience, that is by receiving tactile stimulations all over its artificial skin, including multi-touch stimulations.

### 2.3. Sensorimotor Simulations and Predictive Processes

A growing number of scientists now consider the brain as an active organ of inference (De Ridder et al., 2013; Picard and Friston, 2014; Kirchhoff, 2018). Self-awareness and self recognition are thought to be dependent also on predictive processes - or sensorimotor simulations—implemented by the brain (Hohwy, 2013; Apps and Tsakiris, 2014; Friston, 2018). Predictive processes may have several functions, but one important is that of sensory attenuation. Pyasik et al. (2019) showed that felt ownership of a fake hand in the rubber hand illusion experiment caused attenuation of somatosensory stimuli generated by its movements comparable to the attenuation of self-generated stimuli. Burin et al. (2018) also investigated the influence of timing on the effect of agency.

**Figure 2:**
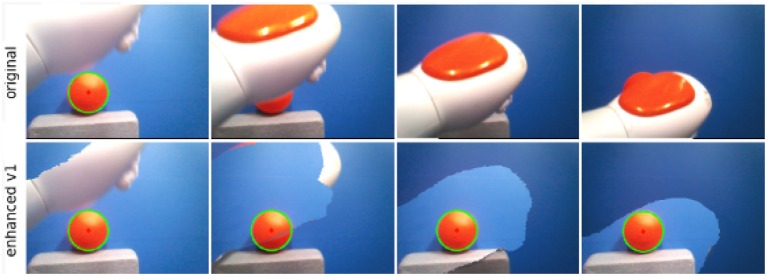
Self-body attenuation through predictive processes (Lang et al., 2018). A humanoid robot Nao is moving its arm in front of an object. The first row shows the frames recorded from its camera. The second row shows the enhanced frames, where self-body perception is attenuated. The attenuation is aided by a forward model, which anticipates the pixels where the robot arm will be visualized, after executing an intended motor command.

**Figure 3:**
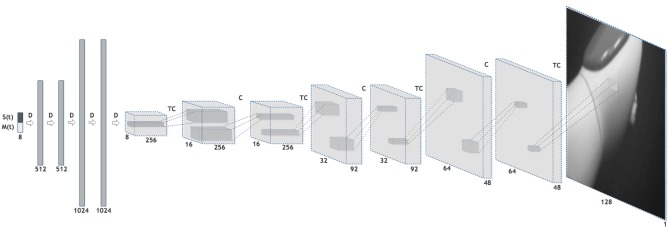
An illustration of the forward model adopted in Lang et al. (2018) for generating image predictions from low-dimensional proprioceptive and motor states through a convolutional neural network. Legend: S(t): sensory state at time t. M(t): Motor command sent at time t. D: Dense, i.e., fully connected, neural network layer. C: Convolutional neural network layer. TC: Transposed Convolutional neural network layer. Every layer except the last (output) one is followed by a ReLU activation unit (not shown) (Lang et al., 2018).

Similar computational models can be implemented into robots to provide them with predictive capabilities. Sensorimotor predictions and prediction errors can be recorded and analyzed, as well. In humans – in contrast – such properties cannot directly be observed and controlled. Bechtle et al. (2016) and Lang et al. (2018) implemented internal models into a humanoid robot to study how body representations can emerge from sensorimotor experience, and how predictive processes can be run through these computational tools. They found that prediction errors can serve as a cue to distinguish between self-generated perceptual events and those generated by other subjects. Moreover, they showed how predictive processes can be used to attenuate self-body perception (see Figure 2). Lang et al. (2018) adopted a convolutional neural network for implementing a forward model, which generates image predictions from low-dimensional proprioceptive and motor states (see Figure 3).

Pico et al. (2016) demonstrated that a two-wheeled mobile robot was capable of detecting unexpected changes in the environment and able to classify motor behaviors by comparing the ego-noise generated by its motors with the ego-noise prediction of its internal model. In a first experiment, several ego-noise prediction models have been trained, each of them with a different motor command pattern. All models were then fed with a particular motor sequence, obtaining a series of ego-noise predictions. The robot was able to determine the correct motor command pattern by selecting the model with the lowest ego-noise prediction error. In a second experiment, one ego-noise forward model has been trained by implementing random motor babbling on the robot in a flat arena. The model was tested by making the robot do a series of runs from side to side of the arena while calculating ego-noise predictions. A ramp was then added in the middle and the runs were repeated. A comparison between the ego-noise prediction errors generated in the flat arena and those of the arena with the ramp on the middle, showed that the ego-noise prediction error increased when the robot was over the ramp. This demonstrated that the robot was able to detect changes in the inclination of the surface it moves only by making ego-noise predictions.

Predictive models can also be used for robot imitation. Pico et al. (2017) utilized robot ego-noise as a mean for communicating intended actions among robots. In an experiment, a robot generated a series of ego-noise audio (emulated by a loudspeaker) representing an intended motor command sequence and conveyed it to another robot. The receiver robot obtained auditory features from the ego-noise through a convolutional autoencoder. These audio features were then fed into an inverse model in order to obtain motor command predictions, which were similar to the motor commands that generated the audio produced by the sender robot.

Winfield (2018) describes a range of different experiments with artificial agents running internal simulations of themselves, others, and the environment, and compares these skills to an artificial Theory of Mind. “Theory of mind is the term given by philosophers and psychologists for the ability to form a predictive model of self and others” Winfield (2018). These internal simulations show how to increase robot safety (Blum et al., 2018) by anticipating self and other behavior (Winfield and Hafner, 2018).

Predictive processes have also been studied by Hinz et al. (2018) in the context of the rubber hand illusion. The authors analyzed the drift in the perception of the real limb toward the fake limb, which would suggest an update of body estimation resulting from stimulation. In particular, they compared body limb drifting patterns of human participants with the end-effector estimation displacement of a multisensory robotic arm enabled with predictive processing perception. They observed similar drifting patterns in both human and robot experiments, suggesting that the perceptual drift is due to prediction error fusion, rather than hypothesis selection.

Touch seems to be a more direct sense, which could be trusted more for prediction than distant senses such as vision. It also equally concerns sense of agency and sense of body ownership. Ciaunica (2019) emphasizes the developmental aspects of touch, self-touch and intersubjective touch. An interesting aspect of predicting the sensory consequences of touch is the feeling of ticklishness, that has been addressed by Sarah Blakemore in a paper with the title “Why can't you tickle yourself” (Blakemore et al., 2000). This phenomenon of ticklishness has also been shown in mice recently (Ishiyama and Brecht, 2016). In a preliminary study on touch prediction in artificial systems, Stiehler and Hafner (2017) could show how a predictive model learns to predict the sensory consequences of touch. The sensory consequences of self-touch are usually more predictable than those of being touched by someone else. The sensation of ticklishness might be triggered by specific changes in prediction error over time, but there is little work so far on this topic. Quantitative studies showed that self-generated forces are perceived in the tactile modality as weaker than externally generated forces of the same magnitude, suggesting again that sensory consequences of a movement are anticipated and attenuated (Shergill et al., 2003).

Vicente et al. (2016) showed how predictive process can also support adaptation of body schemas. The authors combined predictions made by a learned internal model with the actual visual feedback to improve the perceptual skill of a humanoid robot.

The aforementioned studies suggest that predictive processes—as simulations of sensorimotor activities—are important tools for implementing basic cognitive capabilities in artificial systems, and may represent necessary building blocks for providing robots with subjective experiences, such as those typical of the minimal self.

## 3. Metrics for an Artificial Self

As mentioned before, the minimal self is often described by two major building blocks: a sense of body ownership and a sense of agency. Both are subjective measures (articulated by the word “sense”), and can vary between individuals, over time, and depending on the situation. As has been shown in various experiments, for example in the rubber hand illusion (Botvinick and Cohen, 1998), and in virtual reality studies (Blanke and Metzinger, 2009; Banakou et al., 2018), both the sense of body ownership and the sense of agency can be altered in humans. This points toward a certain plasticity of the brain's body representation. Predictive capabilities play a major role in maintaining a consistent minimal self. Based on our self-models, we as humans anticipate the effects of our own actions and can thus monitor them. Longo et al. (2008) for example take a psychometric approach to the question of embodiment and sense of agency based on introspective reports of the rubber hand illusion.

In artificial agents, a similar measure for a sense of body ownership and a sense of agency might be identified. As discussed in the previous sections, most models related to agency and ownership rely on forward models and internal simulations, and have permanent access to a prediction error. When such a model is embodied in an artificial agent, the agent has also direct access to this measure. Michel et al. (2004), for instance, showed in a robotics study that extensions of the self in the visual field can be identified by learning the time delay between actions and their effects.

What could be the necessary requirements of measuring self-ness in artificial agents? In analogy to prediction and anticipation in the human minimal self, a sense of agency and a sense of body ownership should be linked to changes in the prediction error in artificial agents over time as well. Preliminarily ignoring the complex dynamics of the prediction error, we could say that the lower the error in the prediction of the consequences of self-generated actions, the stronger a sense of agency and body ownership.

Given the considerations taken above, we can characterize a self-manifold in sensorimotor space with the following properties: It is dynamic, as it can change with body growth and the acquisition of new skills; it is adaptive, where the error tolerance can vary according to the specific context and the states of the system and of the surrounding environment.

The self-manifold outlines the boundaries of the self, both related to body ownership and agency, which cannot be clearly separated. A concrete example of learning manifolds in sensorimotor space, however not related to the concept of self, can be found in Laflaquière et al. (2015). The boundaries of the self related to body ownership are closely related to notions of peripersonal space (PPS) (Clery and Hamed, 2018). The same can hold for agency if we consider multisensory channels including tactile information and assume temporal and cross-modal predictions (Clery and Hamed, 2018).

Prediction errors—such as those produced by forward models—may be used for determining the boundaries of the self-manifold in the sensorimotor space of artificial agents. Hereby, we encourage further robotics investigation within this research line, as it may provide insights in the understanding of the human self and in the implementation of the artificial self.

This idea follows the argument of Gallese and Sinigaglia (2010) who envision the bodily self as a manifold of action possibilities that cannot be reduced to any form of proprioceptive awareness. Action possibilities necessarily require a system that is able to make predictions about the consequences of own actions. Actions not only include physical body movements and change of postures, but also interaction with the external world, including interaction with objects but also other agents (see Neisser, 1995's notion of interpersonal self).

For simplification, we only consider prediction errors caused by actions affecting the peripersonal space of the agent. A self-metric for an artificial agent is a systematic way to assign a value to each suitable instance of an agent self. It should allow us to compare the self-ness of one agent at a certain instant in time to the self-ness of another agent or the same agent at another instant in time.

Nonetheless, there are still open issues that need to be solved for deciding on such a metric: what timing issues arise; what are the modalities to include or exclude; and which are suitable computational models for multimodal integration. Such a metric will also allow to decide the balance of predicted information vs. perceived information and might ultimately shed light on mechanisms of disturbances of the self in humans.

Similarities to the concept of the self-manifold can be found with that of the *markov blanket* (Kirchhoff et al., 2018). Organisms tend to self-organize within a coherent whole, maintaining a boundary that separates their internal states from the external world. A markov blanket has been theoretised as defining the boundaries of such systems in a statistical sense. If taking the theoretical standpoint of the Free Energy Principle, as proposed by Friston (2013) , this would mean that organisms maintain their integrity by minimizing variational free energy (surprise) over their internal states. That is, they maximize evidence for their own models, i.e., their own existence (Kirchhoff et al., 2018). In predictive coding, free energy is associated with prediction errors. The free-energy bound, or markov blanket, can be associated with a prediction error boundary. A self-manifold may thus be formalized as a markov blanket around the sensorimotor states of an agent.

## 4. Conclusions

In this manuscript, we studied the literature on developmental processes for an artificial self. We reviewed a number of works addressing the self in artificial systems and suggesting basic behavioral and computational components that may serve for the implementation of subjective experiences in robots. However, many questions and challenges in the development of an artificial self still remain open.

In section 2, we reviewed the behavioral and computational components necessary to develop an artificial self - inspired by models of the human self - in the three areas “Self-exploration behaviors and artificial curiosity,” “Body representations,” and “Sensorimotor simulations and predictive processes.” These ingredients of an artificial self have been studied extensively in robotics and computational modeling, and will need to be integrated for a full understanding of the self using computational methods.

A common trend in both analytic sciences such as psychology and neuroscience and synthetic sciences such as robotics is to look more into the developmental processes that shape the self. This allows us to identify prerequisites and test existing theories of the self.

In section 3, we pointed out that beside the challenging task of implementing such mechanisms in artificial systems, there is a need for defining and designing metrics for an artificial self. We suggested requirements for such a self-metric and identified properties of a self-manifold as being adaptive and dynamic. Although we are far from establishing whether artificial agents can ever undergo subjective experiences, these metrics may provide support and insights in the investigation of the self, in both robots and humans.

To conclude this review, we suggest a number of open challenges of the artificial self. In particular, there is a need of integrating the three main behavioral and computational components mentioned above: Self-exploration behaviors and artificial curiosity, body representations, and sensorimotor simulations and predictive processes.

Moreover, further investigation is required in addressing the following overall challenges: designing models for multimodal integration in lifelong learning robotics setups; working on a refinement of self-metrics; identifying difference and complementarity between agency and body ownership; realizing the integration of temporal and intentional binding effects within predictive computational models; and resolving synchronization as well as conceptual issues.

In robotics, we can access internal states and inspect sensorimotor and prediction information. However, to what extent can this privileged point of view allow us to *state*—if ever possible—that a robot is undergoing subjective experience? Indeed, there is a need for further debating the possibility of phenomenological experience in artificial systems.

## Author Contributions

VH and GS produced most of the text within this manuscript. PL and AP contributed to section 2, in particular discussing studies on goal-directed exploration (PL) and ego-noise representation and imitation (AP).

### Conflict of Interest

PL was employed by SoftBank Robotics. The remaining authors declare that the research was conducted in the absence of any commercial or financial relationships that could be construed as a potential conflict of interest.
